# Nanomaterials for endometriosis: a multimodal strategy integrating targeted delivery, immunomodulation, and physical ablation

**DOI:** 10.3389/fphar.2026.1953410

**Published:** 2026-10-26

**Authors:** Muhammad Inam, Muhammad Hayat, Fouzia Latif, Bilal Ahmed, Abuzar Osman

**Affiliations:** 1 School of Medicine, Qilu Institute of Technology, Jinan, China; 2 Department of Basic Health Sciences, College of Applied Medical Sciences, Qassim University, Buraydah, Saudi Arabia

**Keywords:** endometriosis, immunomodulation, magnetic hyperthermia, nanocarrier, nanomedicine, photothermal therapy

## Abstract

Endometriosis is a chronic inflammatory condition for which existing treatments, mainly hormonal therapy and surgical intervention, remain insufficient. These approaches are often limited by high recurrence rates, significant side effects, and an inability to effectively target underlying disease mechanisms. This review outlines emerging mechanism-oriented therapeutic strategies that combine targeted drug delivery, immunomodulation, and physical ablation. Advanced delivery systems, such as Lipid nanoparticle polymeric carriers, and nanomicelles, enable selective accumulation of therapeutic agents at endometriotic lesions, improving efficacy while minimizing systemic toxicity. Immunomodulatory strategies pursue to re-establish immune balance by enhancing immune surveillance, driving macrophage polarization toward anti-inflammatory states, and suppressing pro-inflammatory signaling, often through nanocarrier-enabled localized delivery. Furthermore, physical ablation techniques including gold (Au) NPs-mediated photothermal therapy and magnetic hyperthermia offer minimally invasive approaches for precise lesion elimination while preserving adjacent healthy tissues. Collectively, integrating these strategies may advance preclinical efforts to improve disease management, patient stratification, and potentially fertility outcomes, although clinical validation is still required. Despite ongoing challenges in clinical translation, the convergence of nanotechnology, immunology and interventional therapy marks a conceptual shift away from symptom-centered management toward investigational strategies that, pending clinical validation, may confer disease-modifying potential in endometriosis.

## 1 Introduction

Endometriosis is a debilitating, estrogen-sensitive, chronic, and non-communicable gynecological condition affecting a population of around 10 percent of reproductive-aged women and individuals assigned female sex at birth across the world (Taylor et al., 2021; As-Sanie et al., 2025). The condition is a pattern of complex pathophysiology characterized by ectopic implantation and growth of lesions outside the uterine cavity, most commonly on the peritoneum of the pelvis, ovaries, and bowel (Rathod et al., 2024; Cano-Herrera et al., 2024; Zond et al., 2018). It has an infamously heterogeneous clinical manifestation that ranges from no symptoms up to incapacitating chronic pain that affects the pelvis, dysmenorrhea, dyspareunia, and infertility (As-Sanie et al., 2025; Pašalić et al., 2023). Direct healthcare expenditures, repeated procedures, and significant productivity losses create an enormous economic burden. In contrast, the psychosocial cost, such as anxiety, depression, and loss of quality of life, is immeasurably high. Over decades, endometriosis management has followed a dichotomy between hormonal suppression and surgical excision (Watson, 2024; Perrone et al., 2024; Gheorghisan-Galateanu and Gheorghiu, 2019; Vercellini et al., 2014). The current approach to managing the condition is based on hormonal contraceptives, progestins, gonadotropin-releasing hormone (GnRH) agonists and antagonists, and androgenic agents (Britton et al., 2020; Saleh and Taylor, 2023). Such treatments are aimed at inducing a hypoestrogenic condition, which inhibits the growth and activity of eutopic and ectopic endometrium. Although it works well in most patients to relieve them of the pain, it is basic and non-curative, palliative, and loaded with limitations. The most conspicuous weakness is the high symptom recurrence after treatment discontinuation, since the underlying lesions and their pathobiological factors are suppressed but not eliminated (Elizur et al., 2025; Sarsenova et al., 2024). Moreover, hormonal therapies are not only commonly linked to a set of negative side effects, such as mood swings, weight gain, bone mineral depletion, and anovulation, which make them unbearable and contraindicated in a significant portion of patients, especially patients wanting to conceive (Zhang et al., 2021; Schwartz et al., 2025). This general hormonal concept cannot account for the rich biological diversity of endometriosis, which includes different lesion types, unique molecular profiles, and complex immunomodulatory responses and nervous system interactions (Saunders and Horne, 2021; Czubak et al., 2025). Surgery, mostly laparoscopic excision, can provide a definitive histological diagnosis and eradicate visible lesions (Bafort et al., 2020). It can be regarded as the gold standard in patients who have severe pain or organ dysfunction, however not risk-free. Full excision of lesions with deep involvement is technically difficult and carries a risk of damaging neighboring organs, including the bowel, bladder, and ureters (Walton et al., 2023; Schiavenato and Craig, 2010). The overall inadequacy of existing hormonal and surgical paradigms suggests an urgent clinical need for a new generation of therapeutics. This requirement is based on the fact that we have to undergo the most basic change in our perception of endometriosis pathogenesis. The ancient perception of endometriosis as a retrograde menstruation and estrogen-reliant disorder has been replaced by a much more complex concept (Zafar et al., 2025; Mahdi et al., 2023). Endometriosis is currently identified as a systemic, multidimensional disease, with a constellation of interdependent pathophysiological processes: genetic and epigenetic maladaptation of endometrial cells, which confer its survival, adherence, and invasiveness; the highly perturbed immune microenvironment, including inability to provide natural killer cell cytotoxicity, skewed M1 macrophage polarization, and excessive inflammatory cytokine production (Griffiths et al., 2024; Girling, 2024). The future of endometriosis treatment lies in this complex nexus, where it is necessary to move beyond simple hormonal suppression and adopt a multimodal, targeted approach focused on the pathophysiological mechanisms underlying the disease (Chen et al., 2024).

Although the individual components of this strategy have been reviewed previously, an integrated framework that explicitly connects these three modalities through a shared mechanistic rationale and translates this rationale into a testable theranostic platform under the constraint of fertility preservation remains lacking for endometriosis. Rather than simply combining targeted delivery, immunomodulation and physical ablation, this framework positions them as convergent interventions on a shared pathophysiological axis in endometriosis. Each modality plays a distinct role in enhancing lesion access, disrupting local immune tolerance, and promoting lesion debulking with immune priming, while fertility preservation remains an explicit design constraint. We further propose natural-product-derived carbon dots (CDs) as a testable theranostic embodiment of this integrated framework. This review explores the convergence of three distinct synergistic therapeutic pillars precision nanomedicine, immunomodulation, and physical ablation as the next frontier in endometriosis management (Figure 1). It proposes that, if successfully translated, their integration could shift treatment from conventional systemic suppression to localized, mechanism-based intervention, potentially enabling durable disease modification with improved safety and tolerability. This review also discusses the recent advancements and persistent challenges associated with this multimodal approach. This review further explores the future therapeutic potential of carbon quantum dots (CQDs) and their functionalization for endometriosis via localized drug delivery. Owing to their exceptional biocompatibility, tunable photoluminescence, and facile surface functionalization, CQDs are promising candidates for theranostic applications, facilitating simultaneous fluorescence-based lesion detection and targeted photothermal ablation.

**FIGURE 1:**
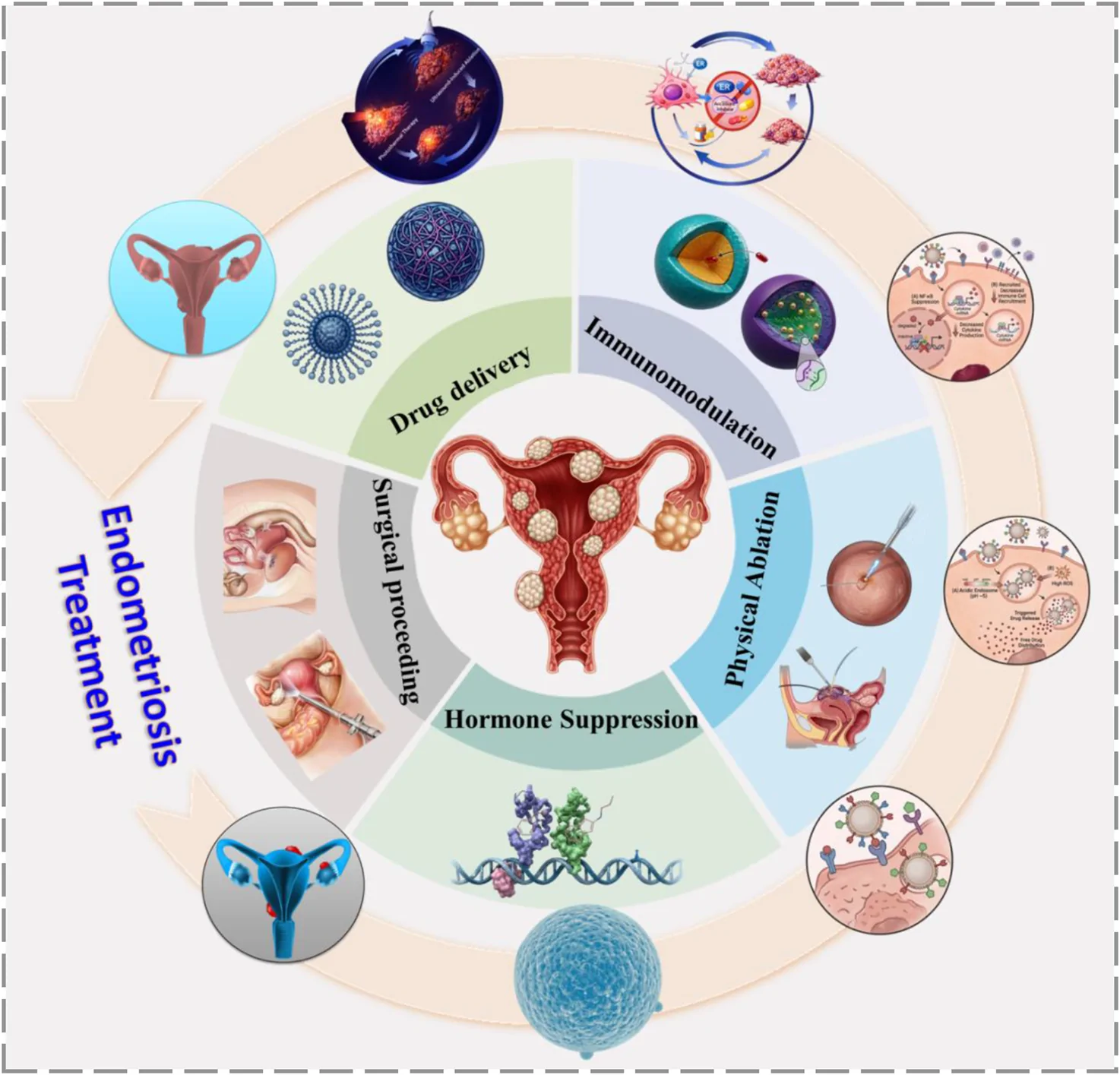
Illustration of endometriosis treatment. The central panel depicts endometrial lesions, encircled by various treatment modalities. The outer circular flow highlights the integrated, combinatorial, and personalized approach to endometriosis management through diverse therapeutic strategies.

## 2 Drug delivery based treatment

Targeted based drug delivery systems for endometriosis are based on the advanced potential of precision nanomedicine that is the most promising solution to the weaknesses of systemic hormonal treatment (Zhang J. et al., 2026). The key principle of nanomedicine is the ability to deliver therapeutic agents with extraordinary long-term and sustained efficacy (Inam et al., 2025). Nanoparticles can selectively accumulate in endometriotic lesions by using a specific targeting ligand on the nanoparticle surface, with particle size and surface charge optimized for the target site (Talukdar et al., 2024; Zhang Q. et al., 2026). This may be done through two major approaches, the passive approach, which takes advantage of the hyperpermeability and retention (EPR) effect of the hyperpermeable neovasculature of active lesions, and the active approach, which involves ligands targeting overexpressed receptors on endometriotic cells, including the folate receptor, integrins (Attia et al., 2019), or selective growth factor receptors (Mansour et al., 2021). Among the wide range of nanocarriers, three platforms have become especially promising for endometriosis treatment: lipid-based nanoparticles, polymer-based systems, and nanomicelles (Figure 2A).

**FIGURE 2:**
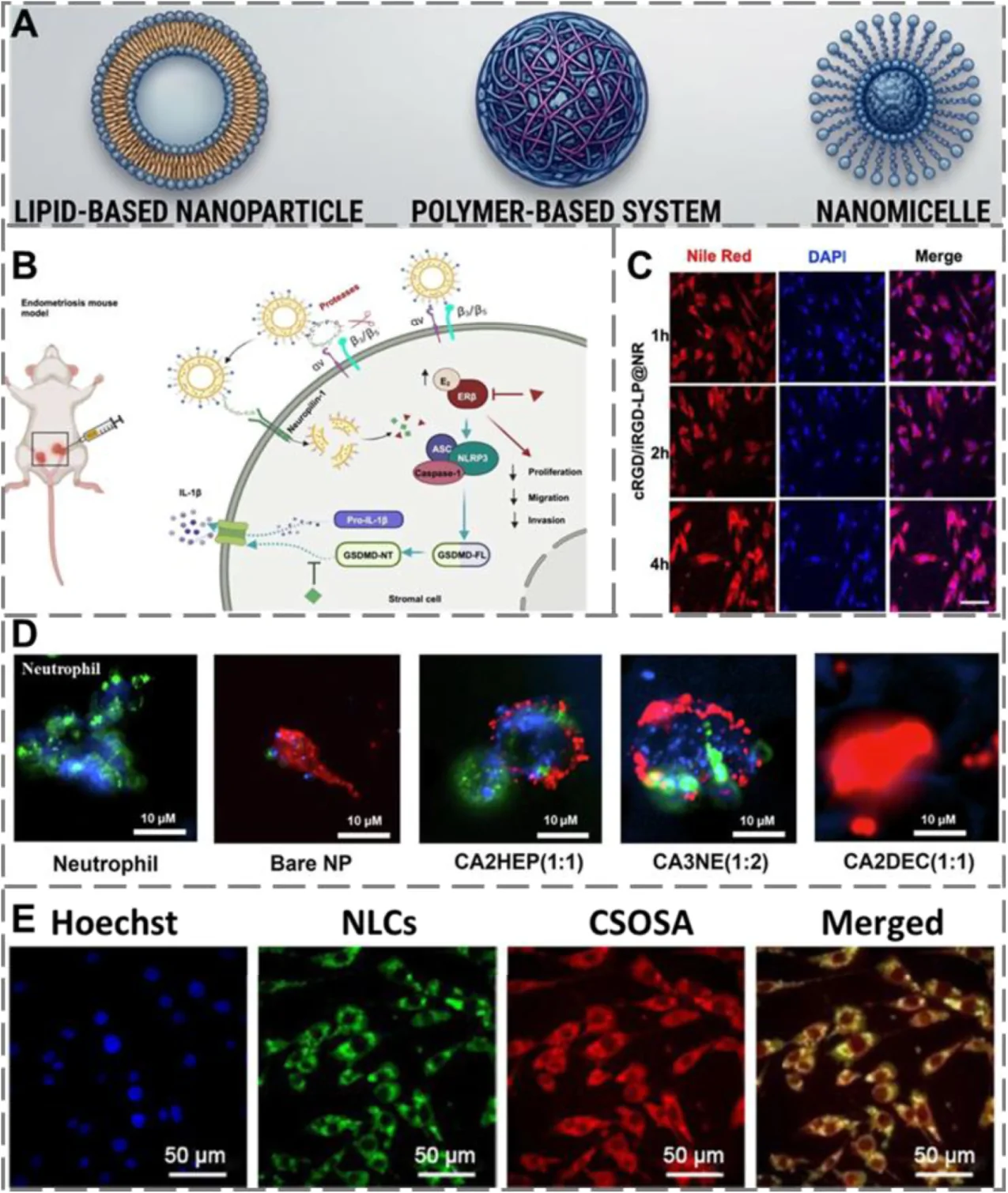
**(A)** Comparative nano platform for endometriosis disease treatment. **(B)** A dual-targeted liposomal nanosystem for the co-delivery of an estrogen receptor β antagonist and disulfiram as a non-hormonal therapy for endometriosis and **(C)** Observation of the uptake of cRGD/iRGD-LP@NR in EESC under CLSM, reproduced from reference (Liao et al., 2025). Copyright©2025 Acta Materialia Inc. Published by Elsevier Inc. **(D)** Live cell confocal images of Au-PLGAIL NPs engaging on the surface of, or uptaken by, neutrophils, reproduced from reference (Vashisth et al., 2026). Copyright©2026, The Author(s) and **(E)** Fluorescence images of CSOSA/NLC internalization in PC12 cells at 12 h, reproduced from reference (Yuan et al., 2017). Copyright©2017, The Author(s).

EPR is highly heterogeneous among patients and lesions, and its magnitude in human disease is typically weaker and more variable than in rodent models. Endometriotic lesions are often small, fibrotic, and poorly vascularized, with nonuniform permeability, elevated interstitial pressure, and vascular characteristics that vary with hormonal and inflammatory status (Khan et al., 2026). Impaired lymphatic drainage may also be inconsistent in peritoneal deposits. Together with interspecies differences in lesion architecture and vascular biology, these features preclude direct extrapolation from animal models (Rockson et al., 2018). EPR should therefore be considered a variable contributor to passive accumulation rather than a reliable targeting mechanism, strengthening the rationale for ligand-mediated targeting and imaging-based patient stratification before EPR-dependent therapy (Dwivedi et al., 2026). Importantly, the EPR effect was established primarily in solid tumors, in which vascular permeability and interstitial pressure differ markedly from those in endometriotic lesions. Direct evidence of EPR-mediated NPs accumulation in human endometriosis remains limited. This distinction matters because endometriotic lesions are generally smaller, more fibrotic, and less vascularized, with permeability governed largely by hormonal and inflammatory status rather than tumor-like angiogenic drive (Vagena et al., 2025).

### 2.1 Lipid-based nanoparticles

Lipid-based nanoparticles (LNPs) are the most clinically developed group of drug delivery systems, due to their high biocompatibility, capacity to entrap both hydrophobic and hydrophilic drugs, and a successful history of clinical use, most recently through the success of mRNA vaccines predominantly in infectious disease and oncology contexts (Hou et al., 2021; Xu et al., 2025). In endometriosis, LNPs provide a flexible platform for targeted therapy (Nomani et al., 2025). The most well-known LNPs are coated with polymers to extend circulation time and use the EPR effect to accumulate passively in hypervascularised endometriotic lesions (Dilliar et al., 2023). Other advanced methods include functionalizing liposomal surfaces with targeting moieties (Riaz et al., 2018). LNPs can co-load multiple therapeutic agents, such as a hormone antagonist and a pro-apoptotic siRNA, positioning them as a potent combinatoric therapy by delivering multiple agents through a single targeted platform (Li et al., 2022). Liao *et al*. developed a targeted liposomal nanosystem (cRGD/iRGD-LP@DP) co-loaded with the pyroptosis inhibitor disulfiram and the estrogen receptor β (ERβ) antagonist PHTPP to synergistically disrupt the positive feedback loop between inflammation and estrogen (Liao et al., 2025). The nanocarrier is modified with the dual-targeting peptides cRGD and iRGD, enabling specific binding to integrin αvβ3 receptors, which are highly expressed in lesions, while leveraging iRGD’s penetrating function to enhance drug delivery into deep tissues (Figures 2B,C). In another study, Abbasi et al. proposed a strategy to deliver therapeutic messenger RNA to the endometrium as a treatment for reproductive disorders (Abbasi et al., 2026). In this approach, mRNA was encapsulated within ligand-conjugated lipid LNPs, facilitating multivalent binding to integrin receptors that are temporally overexpressed on the endometrial surface during the window of implantation. Direct conjugation of the targeting ligand to the lipid moiety enhanced endometrial protein expression following intrauterine infusion while minimizing off-target expression in the liver and spleen. Importantly, this targets the eutopic endometrium in reproductive disorders, not ectopic endometriotic lesions, and therefore represents a related but distinct application. Direct evidence for mRNA–LNP delivery to endometriotic lesions remains lacking, its relevance to endometriosis-associated pain is inferential rather than established. This finding represents a promising therapeutic strategy for the management of pain associated with endometriosis.

### 2.2 Polymer-based nanoparticles

Polymer-based nanoparticles offer an alternative to lipid-based systems, providing structural versatility, long-term release kinetics, and stimuli-responsive drug delivery (El and taib, 2025). Biodegradable polymers, e.g., poly (lactic-co-glycolic acid) (PLGA), have been widely explored for gynecological applications (Nshimiyimana et al., 2025). In particular, PLGA nanoparticles have received special attention because they are FDA-approved, tunable for degradation, and capable of sustained drug release lasting days to weeks (Stevanović et al., 2025). For endometriosis, PLGA nanoparticles are designed to deliver GnRH agonists locally, which may eliminate systemic side effects when administered systemically (Bazybek et al., 2022). Vashisth *et al.* developed ionic liquid-coated gold-core polymeric nanoparticles (Au-PLGA-IL NPs) for selective neutrophil targeting toward photothermal treatment of endometriosis (Vashisth et al., 2026). These nanoparticles demonstrated *in vitro* biocompatibility and *ex vivo* neutrophil selectivity in human whole blood, as confirmed by flow cytometry and confocal microscopy (Figure 2D). This work introduces a novel platform uniquely combining gold plasmonics, PLGA biocompatibility, and the functional versatility of ionic liquids for targeted endometriosis therapy. Surgical intervention effectively removes endometriotic lesions; recurrence often necessitates multiple procedures, driving interest in non-surgical alternatives (Vashisth et al., 2026). Anti-angiogenic therapy using siRNA to target VEGFA expression is a promising approach, with peptide-based polymers offering biocompatible, modifiable delivery platforms (Egorova et al., 2019). Egorova *et al.* evaluated the R6p-cRGD peptide carrier as a non-viral siRNA delivery vehicle to endothelial cells *in vitro* and endometrial implants *in vivo* by assessing complex physicochemical properties, cellular toxicity, and silencing efficiency of GFP and VEGFA genes (Egorova et al., 2024). Peptide-based polymers offer a promising siRNA delivery platform because of their biocompatibility and ease of modification.

### 2.3 Nano-micelles

Micelles are especially suitable due to their small size for passive targeting using the EPR effect (Vagena et al., 2025), since their small size is able to extravasate through the fenestrated vasculature of endometriotic lesions effectively (Moses et al., 2021). Micelles offer an optimal solubilizing environment due to their hydrophobic core, which is a good solvent for poorly water-soluble therapeutic agents, which make up a large percentage of potential anti-endometriotic agents (Hwang et al., 2020). Zhao *et al.* developed a chitosan oligosaccharide-stearic acid polymeric micelle system for gene delivery to overcome limitations of current endometriosis therapies. The cationic nanocomplexes enabled selective targeting of ectopic lesions after intravenous administration in a rat model, reduced lesion size, induced endometrial degeneration, increased apoptosis, and decreased microvessel density without reproductive toxicity, representing a promising therapeutic strategy for endometriosis (Zhao et al., 2012). In addition to passive targeting, active targeting ligands can be functionalized to the micelles (Zhou et al., 2018; Ibrahim et al., 2023). Folate-conjugated micelles have been designed to recognize folate receptor-α, which is highly overexpressed on endometriotic epithelial cells (Senol et al., 2015). Yuan *et al.* developed chitosan oligosaccharide-g-stearic acid (CSOSA) micelle-coated nanostructured lipid carriers (NLCs) as a novel delivery system for the selective P2X3 receptor antagonist A-317491 for endometriosis pain therapy (Yuan et al., 2017). A-317491-loaded NLCs coated with CSOSA micelles formed CSOSA/NLC/A-317491 nanoparticles, which partially inhibited ATP-induced Ca^2+^ influx in P2X3 receptor-expressing PC12 cells. Following intravenous administration in mouse and rat endometriosis models, CSOSA/NLC accumulated in endometriotic lesions. In endometriotic rats, CSOSA/NLC/A-317491 produced prolonged reversal of mechanical and thermal hyperalgesia, likely due to its substantial distribution within the lesions (Figure 2E). Jannatifar *et al.* reported in a randomized trial of endometriosis patients that nanomicelle curcumin supplementation significantly elevated serum antioxidant levels while reducing follicular fluid oxidative stress and pro-inflammatory cytokines (Jannatifar et al., 2025). These changes correlated with improved oocyte yield, embryo quality, and ART outcomes, suggesting that nanomicelle curcumin may be a promising adjunctive therapy for endometriosis-associated infertility.

## 3 Immunomodulation-based treatment

Immunomodulatory nanoparticles are an immune-based intervention approach in endometriosis (do Rêgo and Araújo- Filho, 2025; Dai et al., 2025). Rather than delivering free immunomodulatory agents, which circulate throughout the body and risk off-target immunosuppression, nanoparticle-based delivery enables targeted regulation of selected immune cell populations within the endometriotic microenvironment (Aljabali et al., 2024; Kamothi et al., 2025). Nanoparticles can be designed to directly transfer immunomodulatory payloads to dysfunctional immune cells (Aljabali et al., 2024; Kamothi et al., 2025; Xu et al., 2022). To illustrate, liposomal formulations containing agents of macrophage polarization to induce the anti-inflammatory M2 phenotype, or pro-inflammatory inhibitors to induce the M1 phenotype, have been promising in preclinical trials (Abdollahi et al., 2023; Inam et al., 2026). With a direct delivery of such agents to the macrophages that infiltrate the endometriotic lesions, it can be feasible to re-establish the balance from a pro-inflammatory and disease-promoting state to a pro-resolving and tissue-healing state (Ji et al., 2025). Similarly, nanoparticles carrying immunostimulatory agents can be used to stimulate cytotoxic immunity against endometriotic cells, potentially reversing disease-related immune exhaustion (do Rêgo and Araújo-Filho, 2025; Suszczyk et al., 2024). New therapeutic options have emerged with the realization that endometriotic lesions can use immune checkpoint pathways to avoid macrophage phagocytosis (Chen et al., 2023a; Wang et al., 2025). Direct delivery of nanoparticles functionalized with antibodies or small-molecule inhibitors may deliver agents directly to lesions to restore immune-mediated ectopic cell clearance (Chen et al., 2022; Chen et al., 2018). By enabling the targeted delivery of therapeutic agents to specific immune cells in the endometriotic microenvironment, immunomodulatory nanoparticles offer a precise strategy to restore immune balance and enhance lesion clearance without the off-target effects associated with systemic immunomodulation.

The apparent contradiction between promoting M1-and M2-like macrophage responses reflects context-dependent therapeutic goals. In endometriotic lesions, M2-like macrophages are often co-opted to suppress immunity and support lesion survival, angiogenesis, and fibrosis; reprogramming them toward an M1-like phenotype can restore phagocytosis, antigen presentation, and immune clearance. Conversely, after lesion control, promoting a genuinely pro-resolving M2 phenotype may limit persistent inflammation, tissue injury and adhesion formation. Thus, the objective is not M1 or M2 polarization *per se*, but restoration of stage-appropriate immune balance.

### 3.1 Polymeric micelles in immunomodulation

Polymeric micelles (Figure 3A) have several unique properties that can be used for immunomodulation, especially their ability to target specific immune cell subsets and deliver cargo that regulates immune activity (Moura et al., 2019). The small size and surface versatility of micelles facilitate targeting antigen-presenting cells, e.g., dendritic cells and macrophages, which are key participants in the immune response in endometriosis (Chen et al., 2023a; Amidifar et al., 2025). Micelles are a promising way to deliver immunomodulatory agents to peritoneal macrophages, which are central to endometriosis pathogenesis (Bauer et al., 2021; Costa et al., 2021). Mannose- or other ligand-functionalized versions of these micelles can be selectively taken up by macrophage surface receptors, allowing them to deliver payloads that shift macrophage phenotype from a pro-inflammatory M1 phenotype to a pro-resolving M2 phenotype (Paurević et al., 2024). Immunomodulatory micelles used alongside other therapeutic modalities are of particular interest. For example, co-loaded micelles of an anti-inflammatory agent and a pro-apoptotic agent can suppress the inflammatory microenvironment and induce lesion cell death simultaneously (Panagi et al., 2022). Wu et *al*. developed OMV-coated PLGA nanoparticles as a potential therapy for endometriosis (Wu et al., 2025). These nanoparticles enhanced macrophage internalization while retaining immunostimulatory activity. In mice, the nanoparticles achieved prolonged peritoneal retention and were taken up by nearly 80% of peritoneal macrophages, driving reprogramming toward the M1 phenotype and reducing the M2-to-M1 ratio in both the peritoneal cavity and endometriotic lesions (Figure 3B). Kumar et al. developed a curcumin-loaded mixed micelles formulation and evaluated its compatibility (Kumar et al., 2017). Comparative assessments showed enhanced bioavailability, changes in protein expression, and greater destabilization than control cells (Figure 3C). The findings support further exploration of this formulation to overcome resistance in endometrial cancer. In another study by Zhao *et al.* developed a polymeric micelle gene delivery system using lipid-grafted chitosan micelles (CSO-SA) and pigment epithelium-derived factor (Zhao et al., 2012). Intravenous administration in a rat model enabled targeted delivery of PEDF to endometriotic lesions, significantly reducing lesion size and inducing degeneration without reproductive toxicity. Zhu *et al.* developed a low-cost, scalable glucose oxidase-loaded bovine serum albumin nanoparticle (BSA-GOx-NP). After injection, BSA-GOx-NPs were delivered specifically to ectopic lesions via a neutrophil-dependent mechanism, where they depleted glucose and induced apoptosis (Zhu et al., 2023). Consequently, BSA-GOx-NPs exhibited potent anti-endometriosis efficacy in both acute and chronic inflammatory phases. These findings demonstrate that a neutrophil-hitchhiking strategy is effective in chronic inflammatory disease, offering a non-hormonal and readily accessible approach for endometriosis treatment (Figure 3D). Alternatively, micelles may be formulated to deliver immunomodulatory agents sequentially with physical ablation therapies, with the latter causing immunogenic cell death and the former stimulating the immune system to mount a subsequent anti-lesion response.

**FIGURE 3:**
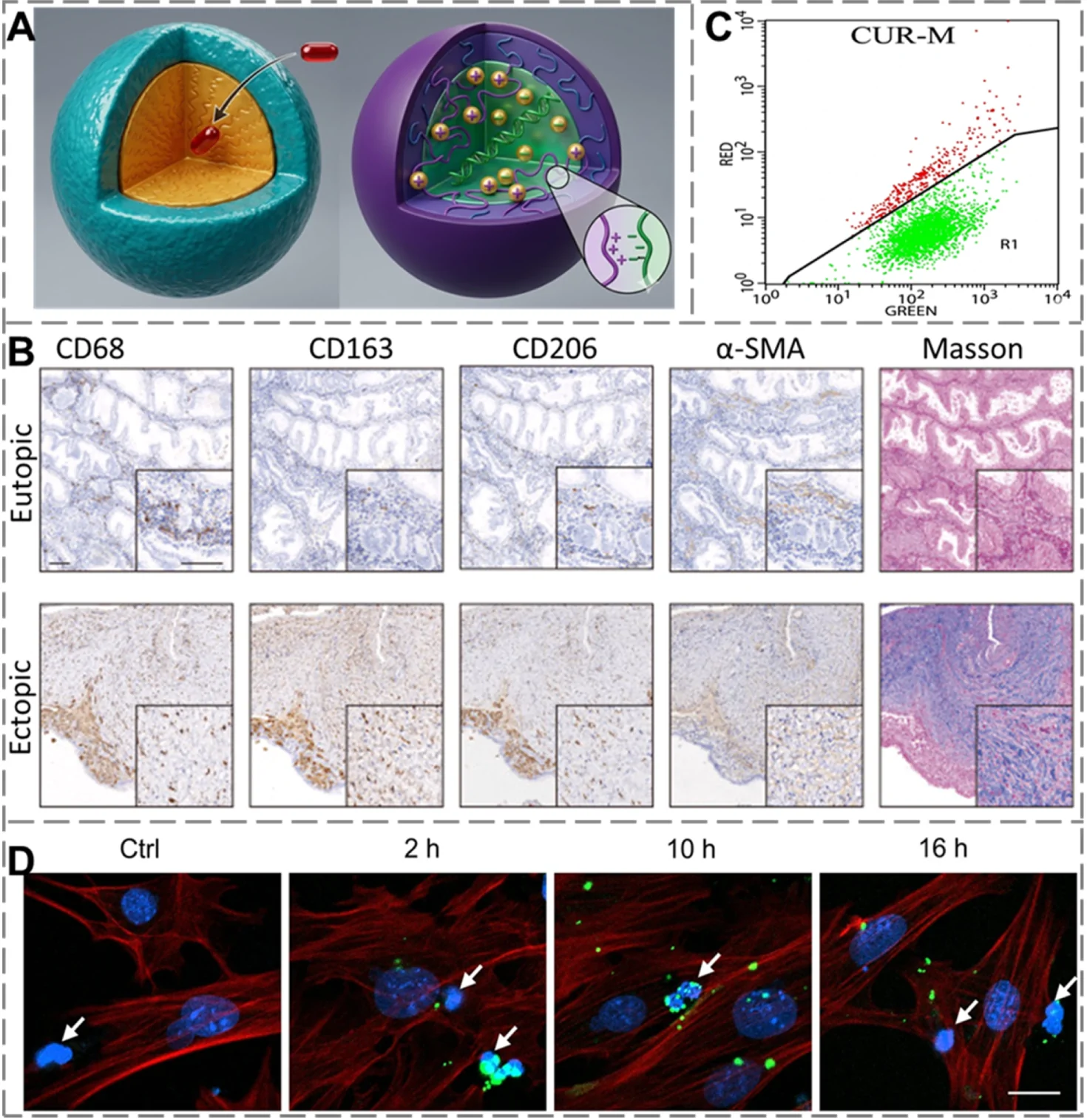
**(A)** Polymeric Micelle Architecture for Immunomodulation in Endometriosis Treatment. **(B)** Following treatment with OMV-coated PLGA nanoparticles, representative immunohistochemical images of CD68, CD163, CD206, and Masson trichrome staining were obtained in paired eutopic and ectopic endometrium, reproduced from reference (Wu et al., 2025). Copyright©2025, The Author(s). **(C)** Effect of curcumin-loaded mixed micellar formulation on mitochondrial membrane potential loss, reproduced from reference (Kumar et al., 2017). Copyright©2017 Elsevier Inc and **(D)** Fluorescence imaging showing the localization of BSA-NPs following co-culture of neutrophils and stromal cells for the indicated time points, reproduced from reference (Zhu et al., 2023). Copyright©2023, The Author(s).

## 4 Physical ablation-based treatment

Image-guided physical ablation-based methods are minimally invasive with the potential of being highly accurate, focused at the location of the endometriotic lesions without causing serious morbidity that traditional surgical excision produces (Bodard et al., 2024). Whereas modalities like high-intensity focused ultrasound (HIFU), laser interstitial thermal therapy (LITT) and microwave ablation (MWA) have already found a place in oncologic ablation, although established in oncology, these modalities remain at an earlier stage of development for endometriosis, with efficacy and safety supported by a relatively limited body of preclinical and clinical evidence with the incorporation of nanotechnology have created two modalities that are currently showing promise in treating endometriosis are AuNPS-mediated PTT and magnetic hyperthermia (Figure 4A) (Park et al., 2022). The methods take the advantage of externally added sources of energy and energy conversion through nanoparticles to cause very localized, controlled tissue destruction (Yuxue et al., 2023).

**FIGURE 4:**
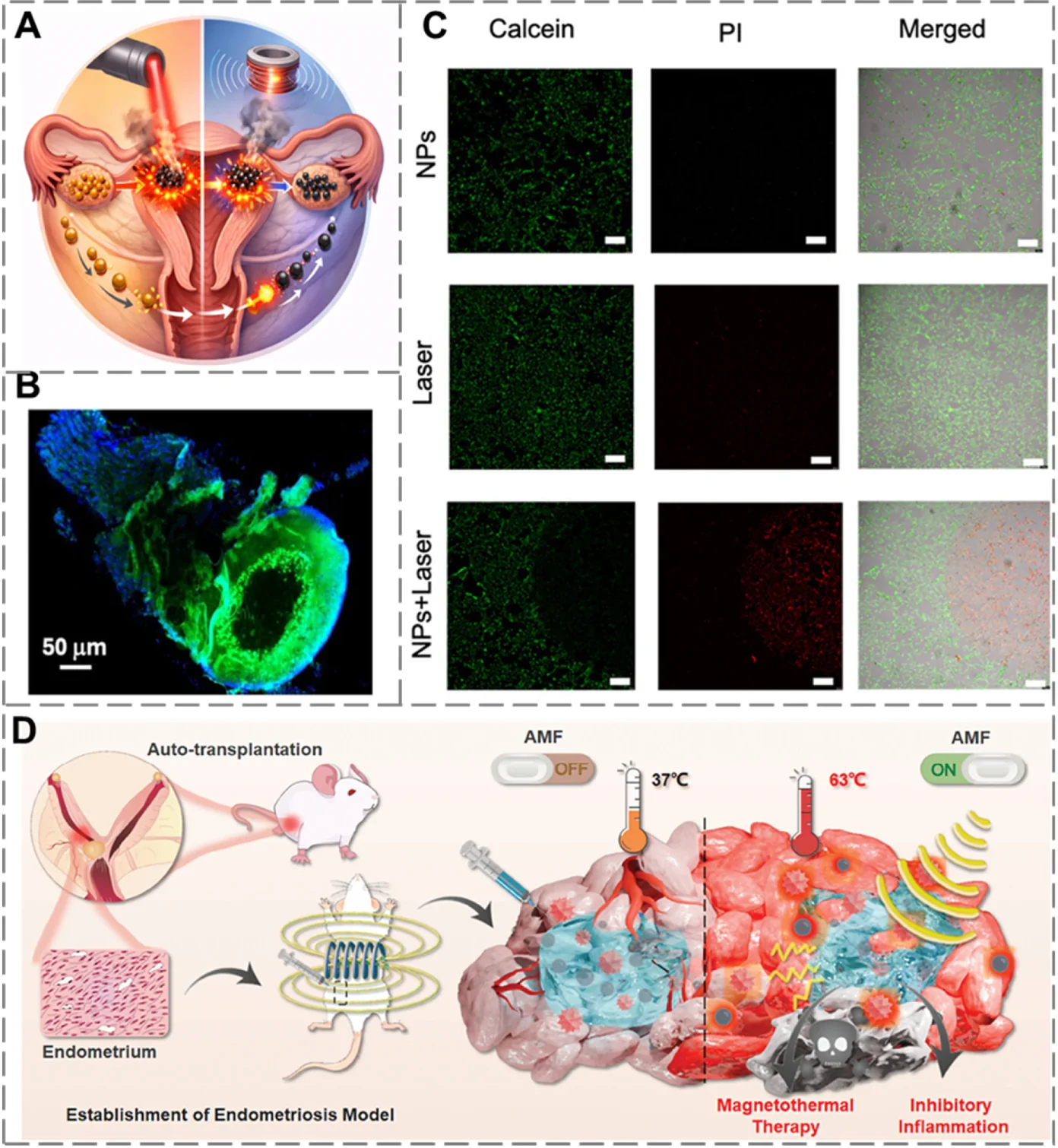
**(A)** Schematic illustration of physical ablation of endometriosis via AuNPs-mediated PTT (left) and magnetic hyperthermia (right). **(B)** Fluorescence microscopy images validating endometriosis lesions following collection from murine models. **(C)** Live/Dead fluorescence images of 12Z cells after photothermal treatment. Reproduced from reference (Kumar et al., 2025). Copyright@2025, The Author(s). **(D)** An Injectable Magnetic Hydrogel Incorporating an Anti-Inflammatory Peptide for Efficient Magnetothermal Therapy of Endometriosis, reproduced from reference (Liu et al., 2024). Copyright@2024, The Author(s).

### 4.1 Gold nanoparticle photothermal therapy

Due to their outstanding optical properties, high biocompatibility, and controllable surface chemistry, gold nanoparticles (AuNPs) have become one of the most versatile and most widely studied platforms of PTT (Amaral et al., 2025). AuNPs PTT for endometriosis takes advantage of the ability to selectively accumulate nanoparticles at lesions and then expose them to NIR light to cause localized thermal ablation (Guo et al., 2017). Gynecological applications have been a special focus for gold nanorods (AuNRs), whose longitudinal surface plasmon resonance can be tuned within the NIR window (Theodorou et al., 2023). AuNRs can be target-ligand functionalized with antibodies against endometrial-specific antigens or peptides against an integrin overexpressed on endometriotic vasculature, to achieve active homing to lesions following intravenous injection (Moses et al., 2021; Tong et al., 2009). This is followed by subcutaneous NIR laser irradiation, delivered using a laparoscopic or percutaneous fiber-optic probe, which rapidly and locally heats the nanoparticle-containing lesions and ablates them with minimal effect on surrounding normal tissues (Pan et al., 2022). NIR irradiation led to complete ablation of the targeted lesions without recurrence after long-term follow-up, and it did not ablate adjacent organs, including the bowel, bladder, and ureters (Fukushima et al., 2022). The absence of adjacent-organ injury in a single preclinical study does not establish clinical safety. Human endometriotic lesions frequently lie adjacent to critical structures, including the bowel, bladder, ureters, major vessels and the risk of thermal injury depends on lesion depth, anatomical proximity, and the precision of light delivery. Clinical safety therefore requires prospective human trials with standardized dosimetry, real-time thermal monitoring, and long-term follow-up. Thermal imaging and MRI thermometry may enable real-time monitoring and controlled modulation of photothermal ablation. However, their reliability in the complex pelvic environment, particularly around mobile organs such as the bowel, remains to be demonstrated clinically (Zhu et al., 2017). In addition to direct thermal ablation, AuNPs PTT may also induce immunomodulatory effects that help maintain long-term disease control. Photothermal ablation can cause immunogenic cell death, triggered by thermal stress, which releases damage-associated molecular patterns (DAMPs) that activate dendritic cells and induce anti-tumor (or anti-lesion) adaptive immune responses. This *in situ* vaccination approach could generate systemic immunity against residual or microscopic endometriotic deposits, addressing a key limitation of focal therapeutic interventions (Riley and Day, 2017). Volpini *et al.* developed a nanosystem based on AuNPs designed to actively target human endometriosis CD44^+^ cells and significantly reduce their viability through photothermal therapy. After internalization via clathrin-mediated endocytosis, the photothermal efficacy was assessed using two laser types, selected based on the nanosystem’s different localized surface plasmon resonance (LSPR) properties. It demonstrated significant photothermal therapeutic efficacy against 12Z cells (Volpini et al., 2025). Kumar *et al.* reported that photoacoustic imaging (PAI) is a noninvasive approach for visualizing deep-seated endometriosis (EM) using FITC-tagged, silica-coated gold nanorods (AuNRs) as contrast agents (Kumar et al., 2025). Upon intravenous injection into EM-bearing mice, pronounced photoacoustic signals originating from the AuNRs were observed in endometriotic tissues, resulting from nanoparticle accumulation within EM lesions driven by the enhanced permeability and retention (EPR) effect. Leveraging their multimodal detection capabilities and therapeutic efficacy, these nanoparticles hold promise for diagnosing, treating, and mechanistically understanding endometriosis (Figures 4B,C). Collectively, these attributes highlight AuNPs-mediated PTT as a promising precision strategy poised to advance the diagnosis, treatment, and long-term management of endometriosis.

### 4.2 Magnetic hyperthermia

Magnetic hyperthermia is an alternative to PTT that uses magnetic nanoparticles, usually iron oxide. Compared to photothermal therapy, in which direct penetration of light is required and the technique is thus limited to lesions that can be visualized via fiber optic probes or laparoscopic view, magnetic hyperthermia has an added benefit of penetrating deeper tissues since the magnetic fields are not notably adversely affected by body tissues (Włodarczyk et al., 2022; Liu et al., 2020). In endometriosis, magnetic hyperthermia offers several unique benefits. First, it can treat deeply infiltrating lesions without direct line-of-sight access, allowing treatment of the uterosacral ligaments, rectovaginal septum, or bowel serosa. Second, magnetic nanoparticles can be administered via multiple routes, such as intraperitoneal administration for diffuse peritoneal disease or intralesional injection for discrete nodules. Third, AMF may be used as an external technique and becomes a truly noninvasive method when combined with a proper targeting strategy (Park et al., 2022; Tan et al., 2025). Magnetic hyperthermia has a therapeutic effect on endometriosis that is confirmed in several preclinical models (Park et al., 2022). In rodent models of endometriosis, studies using SPIONs delivered intraperitoneally or intravenously, followed by exposure to an external alternating magnetic field, have shown substantial lesion regression, decreased production of inflammatory cytokines, and reduced hyperalgesia. Notably, the thermal dose can be directly regulated by manipulating AMF parameters, allowing targeted ablation of specific structures while sparing neighboring vital diagnostic structures (Bruner-Tran et al., 2018). Lui *et al.* have developed an injectable magnetic hydrogel nanosystem designed for the dual application of magnetothermal therapy and anti-inflammatory intervention in endometriosis (Liu et al., 2024). This hydrogel incorporates Fe_3_O_4_ nanoparticles alongside an anti-inflammatory peptide. Upon magnetic activation, the Fe_3_O_4_ nanoparticles induce localized hyperthermia, raising the temperature of endometriotic lesions to 63.3 °C and effectively ablating endometriotic cells. The system demonstrates favorable biocompatibility and complete *in vivo* biodegradability, further supporting its therapeutic potential (Figure 4D). By integrating precise lesion ablation with targeted modulation of the inflammatory milieu, this system presents a novel approach to the clinical management of endometriosis. Magnetic hyperthermia thus emerges as a compelling theranostic approach for endometriosis, combining deep tissue penetrance, spatial selectivity, and multimodal therapeutic capability to address the complex challenges of both superficial and deeply infiltrating lesions.

## 5 Carbon dots as future therapeutics for endometriosis treatment

Carbon dots (CDs) are a promising type of nanomaterial for next-generation therapeutics. CD nanostructures are synthesized from larger quantities of carbon precursors and have a constellation of properties. Their extreme small size allows deep tissue penetration and good cellular uptake (Ross et al., 2020). Moreover, CDs are highly biocompatible, low toxicity, highly photostable and inherently fluorescent, which can be used to provide therapeutic effect and real-time bioimaging, a theranostic capability (Ross et al., 2020). Their high surface area and functional groups serve as anchors for covalent or non-covalent conjugation of various therapeutic agents and enable targeted drug delivery (Singh et al., 2025). In endometriosis, this means CDs can be designed to circulate in the complex peritoneal environment, accumulate in inflamed or ectopic tissues, and provide localized therapy, reducing the whole-body side effects caused by current hormonal therapies. One of the most promising approaches to developing these sophisticated therapeutics is to use natural products as the carbon source and as starting therapeutic agents. Natural compounds have shown great potential in treating endometriosis by targeting a variety of pathways (Tassinari et al., 2023). As a future therapeutic platform, we suggest producing bio-inspired CDs by directly synthesizing natural products known to have anti-endometriotic activity, then co-loading them with a potent analgesic to form a dual-action nanomedicine. As an illustration, carbon dots can be synthesized through a hydrothermal carbonization reaction with retinoic acid as the only precursor (Figure 5). The ensuing natural product-derived CDs would maintain the carbon backbone and could carry surface-bound moieties, which could intrinsically confer bioactivity, e.g., inducing apoptosis and restoring the inflammatory microenvironment of lesions, and also take advantage of the nanoscale properties of CDs to enhance stability and achieve targeted accumulation. These CDs may then be functionalized to accept a non-opioid pain-killing agent. These Nano platforms may be delivered locally. The natural-product-derived CDs component would reduce the size of ectopic lesions by inducing apoptosis, mitigating inflammation, and inhibiting angiogenesis, thereby addressing the anatomical and inflammatory centers of the disease. At the same time, the Pain killer payload would be discharged to activate the dense network by inhibiting the transmission of pain signals at its origin. This synergistic approach simultaneously targets the two most debilitating aspects of endometriosis the disease pathology and the chronic and intractable pain, on a single, biocompatible and theranostic carbon dot platform. This strategy may, if validated, advance the development of a disease-modifying and pain-directed therapy rather than relying solely on symptom-relieving hormonal suppression. At present, however, it remains a conceptual proposal that requires rigorous experimental validation.

**FIGURE 5:**
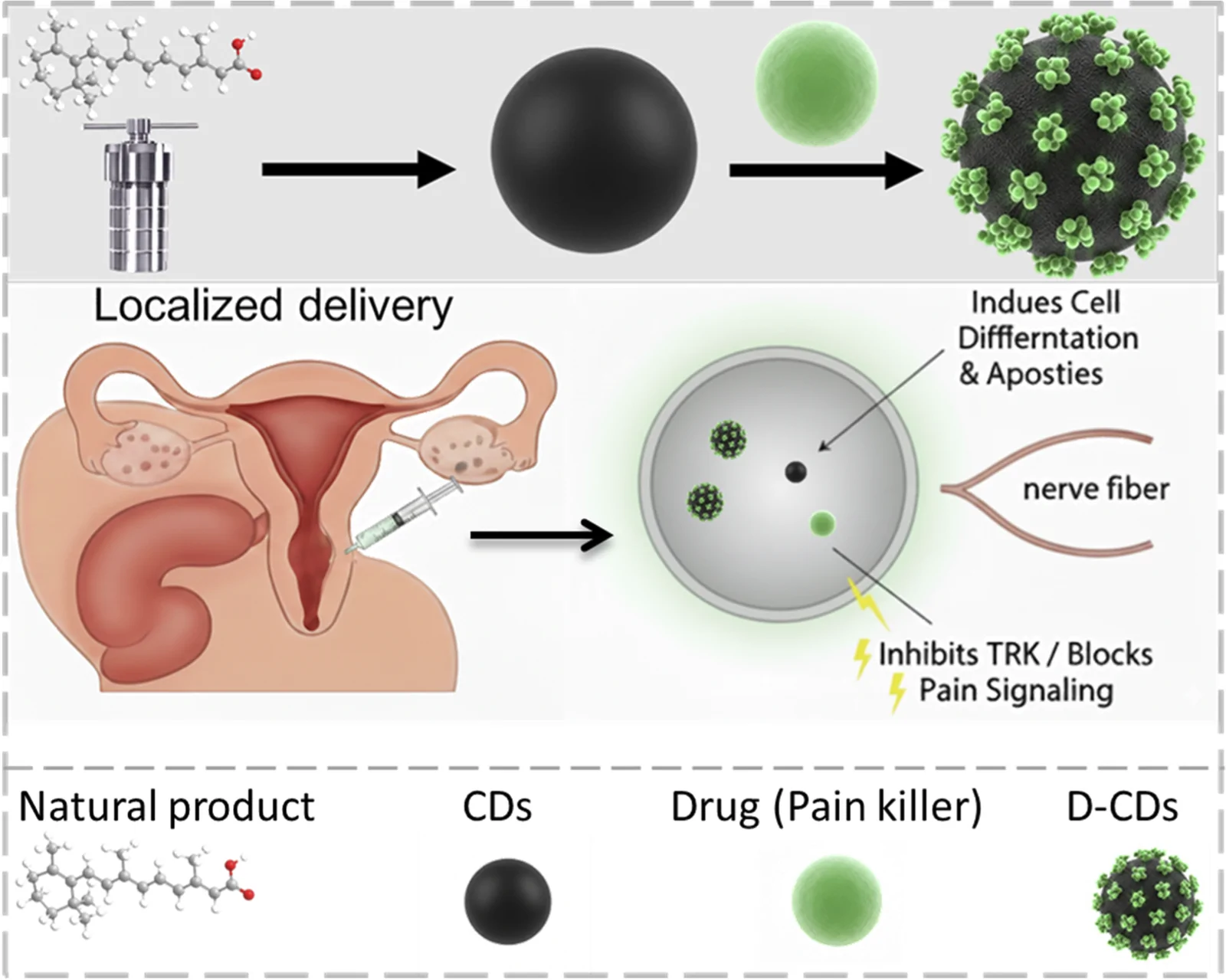
Schematic illustration of natural product–derived carbon dots (CDs) and drug-loaded counterparts (D-CDs) for endometriosis therapy.

## 6 Discussion

The integration of targeted drug delivery, immunomodulation, and physical ablation marks a fundamental shift from palliative hormonal suppression toward mechanism-driven therapy for endometriosis (García-Izquierdo et al., 2024). Targeted drug delivery, immunomodulation, and physical ablation represent a radical transition from palliative hormonal repression to mechanism-based treatment of endometriosis (Brichant et al., 2021; Dolmans and Donnez, 2022). Specifically, active receptor-mediated targeting and passive enhanced permeability and retention (EPR) effects can target endometriotic lesions using delivery systems such as LNPs, polymeric carriers, and nanomicelles, facilitating selective accumulation in diseased tissues, enhancing therapeutic efficacy, and reducing off-target toxicity (Vagena et al., 2025; Slayden et al., 2024). Simultaneously, immunomodulatory strategies that repurpose the dysregulated peritoneal immune context, in particular by converting macrophages from a pro-resolving M2 into a pro-inflammatory M1 phenotype, are the solution to primary immunological triggers of lesion maintenance and progression (Wu et al., 2025; Chen et al., 2023b). Combined with physical ablation modalities, including AuNPs-mediated PTT and magnetic hyperthermia, these strategies can provide localized, image-guided tissue destruction with greater spatial accuracy (Bravo et al., 2024; Nouizi et al., 2024). These synergistic platforms hold potential for durable disease modification, personalized treatment, and improved fertility outcomes, although clinical evidence remains limited. Although these multimodal strategies are encouraging in preclinical settings, significant challenges remain in clinical translation. These include the intrinsic heterogeneity of endometriotic lesions, the lack of reliable noninvasive biomarkers to stratify patients, and the need for strict safety monitoring in the reproductive tract when nanoparticle-based systems are used. Future research should focus on theranostic systems, i.e., functionalized carbon dots, that enable real-time lesion imaging and targeted therapy for individualized intervention (Vordos et al., 2023). Combining the immunomodulatory nanoparticles with focal ablation may harness *in situ* vaccination effects to induce lesion-specific immunity, an approach that warrants investigation as a strategy to prevent disease recurrence (Wei et al., 2023; Zhou et al., 2026; Mao et al., 2020). Multidisciplinary cooperation among nanomedicine engineers, immunologists, interventional radiologists, and reproductive specialists will be necessary to develop standardized protocols, navigate regulatory pathways, and demonstrate long-term safety and efficacy through well-designed clinical trials.

Targeted delivery has the strongest preclinical efficacy evidence but limited long-term safety data, immunomodulation is supported by a strong mechanistic rationale but lacks direct efficacy evidence in endometriosis and physical ablation provides immediate lesion control but may be less durable (Srivastava et al., 2026; Liu et al., 2017). Clinically, targeted delivery is the most advanced, physical ablation is intermediate and immunomodulation remains the least mature (Mansouri et al., 2024). The distinctive contribution of this review is to position these modalities as convergent interventions along a shared pathophysiological axis, explicitly incorporate fertility preservation as a design constraint, and propose natural-product-derived carbon dots as a testable theranostic platform.

## 7 Conclusion

There is a paradigm shift in the management of endometriosis whereby hormonal suppression has been used to manage the condition through symptom suppression, to the use of multimodal therapeutic interventions. A combination of focused nanomedicine, immunomodulation, and physical ablation offers a synergistic model that combats the disease’s complex pathophysiology, enabling targeted lesioning, restoration of immune homeostasis, and precise tissue ablation with organ-sparing accuracy. New technologies, such as functionalized CDs, also show the potential to integrate theranostics through real-time imaging and local therapy. Despite encouraging preclinical developments, clinical translation would require stringent safety testing, biomarkers to stratify patients, and multidisciplinary efforts in nanotechnology, immunology, and interventional radiology. Ultimately, if key translational challenges can be overcome, this combined paradigm may prove transformative by enabling durable disease modification, improved fertility outcomes, and personalized management of endometriosis. At present, however, these approaches should be considered investigational.
